# Postsynaptic Protein Shank3a Deficiency Synergizes with Alzheimer's Disease Neuropathology to Impair Cognitive Performance in the 3xTg-AD Murine Model

**DOI:** 10.1523/JNEUROSCI.1945-22.2023

**Published:** 2023-06-28

**Authors:** Olivier Landry, Arnaud François, Méryl-Farelle Oye Mintsa Mi-Mba, Marie-Therese Traversy, Cyntia Tremblay, Vincent Emond, David A. Bennett, Karen H. Gylys, Joseph D. Buxbaum, Frédéric Calon

**Affiliations:** ^1^Faculté de pharmacie, Université Laval, Quebec G1V 0A6, Quebec, Canada; ^2^Axe Neurosciences, Centre de recherche du CHU de Québec-Université Laval, Quebec G1V 4G2, Quebec, Canada; ^3^Rush Alzheimer's Disease Center, Rush University Medical Center, Chicago, Illinois 60612; ^4^School of Nursing, University of California, Los Angeles, California 90095; ^5^Seaver Autism Center for Research and Treatment, Icahn School of Medicine at Mount Sinai, New York 10029, New York

**Keywords:** aging, Alzheimer's disease, mouse model, neurodegenerative disease, Shank3, synaptic plasticity

## Abstract

Synaptic loss is intrinsically linked to Alzheimer's disease (AD) neuropathology and symptoms, but its direct impact on clinical symptoms remains elusive. The postsynaptic protein Shank3 (SH3 and multiple ankyrin repeat domains) is of particular interest, as the loss of a single allele of the *SHANK3* gene is sufficient to cause profound cognitive symptoms in children. We thus sought to determine whether a SHANK3 deficiency could contribute to the emergence or worsening of AD symptoms and neuropathology. We first found a 30%-50% postmortem loss of SHANK3a associated with cognitive decline in the parietal cortex of individuals with AD. To further probe the role of SHANK3 in AD, we crossed male and female 3xTg-AD mice modelling Aβ and tau pathologies with *Shank3a*-deficient mice (Shank3^Δex4-9^). We observed synergistic deleterious effects of Shank3a deficiency and AD neuropathology on object recognition memory at 9, 12, and 18 months of age and on anxious behavior at 9 and 12 months of age in hemizygous Shank3^Δex4-9^-3xTg-AD mice. In addition to the expected 50% loss of Shank3a, levels of other synaptic proteins, such as PSD-95, drebrin, and homer1, remained unchanged in the parietotemporal cortex of hemizygous Shank3^Δex4-9^ animals. However, Shank3a deficiency increased the levels of soluble Aβ_42_ and human tau at 18 months of age compared with 3xTg-AD mice with normal Shank3 expression. The results of this study in human brain samples and in transgenic mice are consistent with the hypothesis that Shank3 deficiency makes a key contribution to cognitive impairment in AD.

**SIGNIFICANCE STATEMENT** Although the loss of several synaptic proteins has been described in Alzheimer's disease (AD), it remains unclear whether their reduction contributes to clinical symptoms. The results of this study in human samples show lower levels of SHANK3a in AD brain, correlating with cognitive decline. Data gathered in a novel transgenic mouse suggest that Shank3a deficiency synergizes with AD neuropathology to induce cognitive impairment, consistent with a causal role in AD. Therefore, treatment aiming at preserving Shank3 in the aging brain may be beneficial to prevent AD.

## Introduction

Alzheimer's disease (AD) is affecting >40 million people worldwide and is the leading cause of dementia. Tau-laden neurofibrillary tangles and Aβ deposits are the most studied neuropathological hallmarks of the disease (Tremblay et al., 2007; Iqbal et al., 2016; Scheltens et al., 2016). However, evidence gathered from human postmortem studies over the past 20 years indicates that synaptic dysfunction may be an early event in the pathogenesis of AD (Terry et al., 1991; Selkoe, 2002; Scheff et al., 2014; Forner et al., 2017; Tremblay et al., 2017; Jellinger, 2020). Various synaptic proteins have been found in lower amounts in the brain of AD patients, the most consistently investigated being synaptophysin in many (Masliah et al., 2001; Calon et al., 2004; Gylys et al., 2004; Counts et al., 2006; Ishibashi et al., 2006; Julien et al., 2009; Pham et al., 2010; Tremblay et al., 2017), but not all, studies (Honer et al., 2012). Such decreases in synaptic protein levels have been suggested to precede neuronal loss and to correlate with clinical symptoms at least as strongly as Aβ and tau pathologies (Terry et al., 1991; Scheff et al., 2007; Bereczki et al., 2016; Tremblay et al., 2017). The importance of synaptic defects is also supported by data in transgenic animal models of AD neuropathology (Calon et al., 2005; Jacobsen et al., 2006; Bories et al., 2012; Arsenault et al., 2013; Pozueta et al., 2013; Clark et al., 2015). However, since genetic depletion of key synaptic proteins, such as synaptophysin, has limited phenotypic consequences (Eshkind and Leube, 1995; McMahon et al., 1996; Fujiwara et al., 2006; Aramendy et al., 2013; Raja et al., 2019), it remains elusive to what extent the decrease of a synaptic protein is translated into clinically significant effects.

The postsynaptic protein Shank3 (SH3 and multiple ankyrin repeat domains) stands as a notable exception, given that a 50% loss of Shank3 leads to cognitive impairment and possibly neurodegeneration in humans (Grabrucker et al., 2011a, b; Denayer et al., 2012; Phelan and McDermid, 2012; Guilmatre et al., 2014). Shank3 is a scaffold protein central to postsynaptic densities (PSDs) promoting the development of dendritic spines and the formation of excitatory synapses in addition to maintaining the function of glutamate receptors (Naisbitt et al., 1999; Roussignol et al., 2005; Dosemeci et al., 2016). PSDs located on spine heads facing presynaptic boutons are the home of most key postsynaptic proteins, including several subtypes of glutamate receptors and scaffold proteins, such as PSD-95, Drebrin (Developmentally Regulated Brain Protein), and Homer, in close interaction with Shank3 (Sheng and Hoogenraad, 2007; Verpelli et al., 2012). The loss of one copy of *SHANK3* has been identified as a cause for neurologic and behavioral symptoms in children (Wilson et al., 2003a; Bonaglia et al., 2006; Phelan and McDermid, 2012). Consistent with a possible role in neurodegeneration and not just in neurologic development, *SHANK3* defect is suspected to cause symptom aggravation and skill regression in young patients and possibly dementia-like symptoms in older subjects (Denayer et al., 2012; Vucurovic et al., 2012; Guilmatre et al., 2014). The effects of *SHANK3* deletion on synaptic function and social interaction have been replicated in transgenic models (Bozdagi et al., 2010; Peca et al., 2011; Jiang and Ehlers, 2013; Kouser et al., 2013; Jaramillo et al., 2017). Such a demonstrated allelic dose–response effect is unique among all synaptic proteins and argues for a key role of Shank3 in synaptic pathologies underlying cognitive impairment. However, while *SHANK3* has been the subject of extensive research in the field of autism spectrum disorder, its role in age-related cognitive disorders, such as AD, remains unclear (Gong et al., 2009; Pham et al., 2010; Grabrucker et al., 2011b; Guilmatre et al., 2014). In this study, we investigated the relationship between Shank3 deficiency, cognitive impairment, as well as Aβ and tau neuropathologies in brain samples from the Religious Orders Study and in hemizygous 3xTg-AD mice lacking one *Shank3* allele (Shank3^Δex4-9^), assessed at different ages.

## Materials and Methods

### Human brain samples: Religious Orders Study (Rush Alzheimer's Disease Center)

Parietal cortex samples were obtained from participants in the Religious Orders Study, a longitudinal clinical and pathologic cohort study of aging and dementia from which extensive amounts of clinical and neuropathological data were available (Bennett, 2006; Bennett et al., 2012). The study was approved by an Institutional Review Board of Rush University Medical Center. All participants signed an informed consent, Anatomic Gift Act, and a repository consent to allow their materials to be shared. Each participant enrolled without known dementia and underwent uniform structured clinical evaluations until death as previously described (Bennett et al., 2006). A global cognitive score was determined for each subject based on 19 tests that assess a range of cognitive abilities from five cognitive domains, including episodic, semantic, working memory, perceptual speed, and visuospatial ability (Wilson et al., 2003b; Bennett et al., 2012). In the present work, cases were classified using the Braak score based on neurofibrillary tau pathology, either as AD (Stages 4 and 5) or Controls (Stages 1-3). Table 1 summarizes clinical, neuropathological, and biochemical data of participants grouped by Braak-based AD diagnosis (Tremblay et al., 2007, 2017; Bourassa et al., 2019b).

**Table 1.** Cohort characteristics*^a^*

Characteristic	Braak Stages 1-3	Braak Stages 4 and 5	Statistical analysis
*N*	18	18	—
Men, %	27	27	C; Pearson test, χ2 = 0; *p* = 1.0
Mean age at death (yr)	86.0 (4.7)	84.4 (5.7)	Mann–Whitney test, *p* = 0.29
Mean education (yr)	18.3 (3.5)	18.4 (2.8)	Mann–Whitney test, *p* = 0.81
Mean MMSE	25.2 (7.0)	21.8 (7.6)	Mann–Whitney test, *p* = 0.076
Global cognition score	−0.61 (0.96)	−0.94 (0.94)	Mann–Whitney test, *p* = 0.15
apoE ε4 allele carriage (%)	17	56^†^	C; Pearson test, χ2 = 5.90; *p* = 0.015
Reagan score 3/2/1 (*n*)	10/8/0	4/7/7	—
CERAD score 4/3/2/1 (*n*)	5/4/7/2	4/0/5/9	—
ABC diagnosis Ctrl/AD (*n*)	8/10	3/15	—
Clinical diagnosis NCI/MCI/AD (*n*)	8/5/5	4/7/7	—
Cerebellar pH	6.42 (0.31)	6.45 (0.29)	Mann–Whitney test, *p* = 0.68
Postmortem delay (h)	5.26 (3.27)	7.86 (5.82)	Mann–Whitney test, *p* = 0.24
Neuron diffuse plaque counts	7.44 (9.7)	20.6 (19.7)*	Mann–Whitney test, *p* = 0.011
Neuron plaque counts	5.4 (6.9)	15.2 (15.9)*	Mann–Whitney test, *p* = 0.024
Soluble Aβ_40_ concentration (fg/μg protein)	132.7 (59.8)	689.2 (855.1)	Mann–Whitney test, *p* = 0.088
Soluble Aβ_42_ concentration (fg/μg protein)	1497.2 (1536.3)	2772.2 (2312.7)*	Mann–Whitney test, *p* = 0.010
Insoluble Aβ_40_ concentration (pg/mg tissue)	4.70 (7.22)	66.5 (113.7)	Mann–Whitney test, *p* = 0.33
Insoluble Aβ_42_ concentration (pg/mg tissue)	581.3 (652.9)	1507.5 (1169.7)	Mann–Whitney test, *p* = 0.084
Neurofibrillary tangle counts	0.17 (0.51)	4.50 (11.44)	Mann–Whitney test, *p* = 0.082
Total insoluble tau	660.7 (268.2)	2164.2 (1633.2)*	Mann–Whitney test, *p* = 0.0042
Insoluble phospho-tau (S396/404)	24.0 (58.1)	1406.0 (2075.6)***	Mann–Whitney test, *p* = 0.0008

*^a^*Subjects were grouped based on Braak neuropathological assessment as controls (Braak Stages 1-3) or AD (Braak Stages 4 and 5). All neuropathology and biochemistry were performed in the parietal cortex. Soluble Aβ peptide concentrations were determined by ELISA. Tau concentrations were measured using WBs in formic acid extracts with antibodies Tau 13 and PHF1 to assess total tau and insoluble phospho-tau (S396/404), respectively. Brain pH was measured in cerebellum extracts. Values are mean (SD) unless specified otherwise. C, Contingency; CERAD, Consortium to Establish a Registry for Alzheimer's Disease; MCI, mild cognitive impairment; NCI, healthy controls with no cognitive impairment.

Statistical analysis (comparing Braak Stages 4 and 5 to Braak Stages 1-3): Mann–Whitney test,

*p < 0.05,

***p < 0.001; Pearson's χ^2^ contingency test,

^†^*p* < 0.05.

### Human sample preparation

Protein fractions of human parietal cortex samples were obtained by homogenization in TBS (0.05 m Tris-base, 0.138 m NaCl, 2.7 m KCl) containing phosphatase and protease inhibitors (BioTools) and then sequentially centrifuged and sonicated to separate the TBS-soluble fraction (containing intracellular and extracellular soluble proteins) from the detergent-soluble fraction (containing detergent-soluble membrane-bound proteins) by addition of 0.5% sodium deoxycholate, 0.5% SDS, 1% Triton X-100 in the pelleted fraction (Tremblay et al., 2017).

To generate synaptosomes, unfixed fresh samples (∼0.3-5 g) were minced in 0.32 m sucrose the day of autopsy, slowly frozen, and stored at −80°C until homogenization. The crude synaptosomal pellet was prepared as described previously (Gylys et al., 2003; Sokolow et al., 2015) and centrifuged and sonicated to generate TBS-soluble and detergent-soluble fractions, as detailed above. Samples were resolved on a freshly prepared SDS-PAGE, transferred, and blotted with antibodies against tau phosphorylation at epitope S396/404 (paired-helical-filament [PHF1] antibody, gift from Peter Davies; Feinstein Institute for Medical Research), total human tau (tau 13 antibody, Covance/Biolegend), and anti-Shank3 (Abcam, ab136429).

### Generation of Shank3^Δex4-9^-3xTg-AD mice

To study the interrelation between Shank3 deficiency and AD neuropathology, we crossed two transgenic lines. The 3xTg-AD (APPswe, PS1M146V, tauP301L) mouse model expresses mutated human APP and tau and develops an age-related progressive neuropathological phenotype that includes both Aβ plaques and neurofibrillary tangles distributed along a regional pattern similar to AD (Oddo et al., 2003; Vandal et al., 2014). The Shank3^Δex4-9^ mouse is a model of Shank3 deficit obtained by excision of exons 4-9 of the *Shank3* gene, resulting in a constitutive deletion of the Shank3a protein. Shank3a-deficient 3xTg-AD mice were generated by crossing homozygous 3xTg-AD mice with homozygous B6(Cg)-*Shank3^tm1.2Bux^*/J mice (referred to as Shank3^Δex4-9^ in this study) from Mount Sinai School of Medicine (Bozdagi et al., 2010; Yang et al., 2012) to create a new model here referred to as the Shank3^Δex4-9^-3xTg-AD model. Four groups were obtained from the Shank3^Δex4-9^ × 3xTg-AD crossing. The first corresponds to control mice carrying no mutations (*n* = 146). The second group corresponds to hemizygous animals carrying one copy of each 3xTg-AD transgene and normal *Shank3* alleles (*n* = 151). The third group corresponds to hemizygous mice carrying one deletion of *Shank3*^Δ*ex4-9*^ and no 3xTg-AD mutation (*n* = 148). The last group corresponds to the animals carrying one copy of each 3xTg-AD transgene, as well as one Shank3^Δex4-9^ deletion (*n* = 147). Balanced numbers of males (*n* = 72-77) and females (*n* = 72-74) were killed at 4, 6, 9, 12, or 18 months of age for each group.

All mice were put under deep anesthesia with intraperitoneal injections of ketamine/xylazine (90 mg/kg ketamine, 10 mg/kg xylazine) and killed by intracardiac perfusion of 50 ml ice-cold 0.1 m PBS containing phosphatase and protease inhibitors (SigmaFAST proteases and phosphatases inhibitor tablets; Sigma-Aldrich). The brain was rapidly dissected and frozen at −80°C until processing. To generate brain tissue suitable for immunofluorescence experiments, mice were perfused by sequentially passing 0.1 m PBS, acrolein 3.5% (in 0.1 m PB), and PFA 4% (in 0.1 m PB). All experiments were performed in accordance with the Canadian Council on Animal Care and were approved by the Institutional Committee of Université Laval.

### Behavioral tests

The effect of Shank3 deficiency was evaluated using three behavioral tests, as previously described (Arsenault et al., 2011; St-Amour et al., 2014). They were conducted 1 week before death with a recovery time of 24 h between each test. The novel object recognition (NOR) test was developed to study learning and memory in rodents and is based on their spontaneous tendency to have more interactions with a novel than a familiar object (Balderas et al., 2008; Antunes and Biala, 2012). Animals were exposed during 5 min to two objects (familiarization phase). Cognition memory was tested 1 h later by exposing the animal to one familiar and one novel object during 5 min. The Recognition Index (RI) was established as the time spent interacting with the novel object divided by the total time of exploration during the testing phase. Animals for which the exploration time was considered insufficient to allow recognition (<10 s of exploration during the familiarization phase or no exploration of at least one object in the recognition phase) were excluded from analysis.

The light & dark box test (aka dark emergence test) was used to evaluate the anxiety-like behavior. Mice were initially placed in the center of the dark chamber and had free access to the illuminated chamber. The total time spent in the illuminated chamber, the latency of first entry, and the number of alternation between sides were recorded for 5 min. A reduction in the number of alternations or in the time spent in the illuminated compartment was interpreted as increased anxiety. Finally, the open field test measured the general locomotor activity. Movements were tracked by the automated recording of photobeam breaks (San Diego Instruments) to measure activity for 1 h. The ratio between the total time exploring the central area/peripheral area and distance traveled was analyzed. *z* scores were calculated for the latency of the first entry, the number of entries, and the time spent in the illuminated chamber (light & dark box test) and the ratio of time spent in the central area over the peripheral area of the chamber (open field test) separately for each age using the following formula: (individual score – average of all experimental groups)/SD of all experimental groups

Using this method, four avoidance-related variables were reduced to one composite score, allowing a consistent method to assess the anxiety of the mice while avoiding to arbitrarily choose independent variables for effects (Sterniczuk et al., 2010; Toth et al., 2016; Deslauriers et al., 2017, 2019).

### Protein fractionation from brain tissue

The detailed procedure for protein extraction was described previously (Lebbadi et al., 2011). Briefly, half of mouse parietotemporal cortices (∼100 mg) were first homogenized in TBS (0.05 m Tris-base, 0.138 m NaCl, 2.7 m KCl) containing phosphatase and protease inhibitors (BioTools) and then sequentially centrifuged to generate a TBS-soluble fraction (intracellular and extracellular fraction), a detergent-soluble fraction (membrane fraction), and a detergent-insoluble fraction (insoluble aggregated proteins resuspended in formic acid). The detergent-insoluble fraction was separated in two and dried out. A part was resuspended with 5 m guanidine diluted in 0.05 m Tris-HCl to be used for ELISA (see below) and the rest was solubilized in 1× Laemmli's buffer and processed for Western immunoblotting (WB).

The other half of mouse parietotemporal cortices were sequentially centrifuged to obtain a postsynaptic density protein enriched fraction (PSD fraction). The method has been adapted from previous reports (Carlin et al., 1980; Bozdagi et al., 2010; Schmeisser et al., 2012). Briefly, a homogenate was obtained by mechanical mixing with a Potter in Buffer A (4 mm HEPES, pH 7.4; 0.32 m sucrose, phosphatase and protease inhibitors, BioTools). Nuclear fractions were precipitated by centrifuging twice at 700 × *g* for 15 min, and the resulting supernatants were further centrifuged at 16,000 × *g* for 15 min. The pellet was then mechanically mixed with the Potter in Buffer B (4 mm HEPES, pH 7.4, phosphatase and protease inhibitors, Biotools). The sample was then rotated at 4°C for 1 h and centrifuged at 25,000 × *g* for 20 min. The pellet was then resuspended in Buffer C (50 mm HEPES, pH 7.4; 2 mm EDTA, 0.5% Triton X-100, phosphatase and protease inhibitors, Biotools) and agitated 15 min at 4°C. The resuspended pellet was then sequentially centrifuged at 32,000 × *g* and 180,000 × *g* for 20 min at 4°C. Finally, PSD fractions were resuspended in HEPES-C containing 1.8% SDS and 2.5 m urea and stored as −80°C until further use.

### In situ hybridization

*In situ* hybridization was performed according to general methodology, as described previously (Julien et al., 2008, 2009; Delay et al., 2014). DNA oligonucleotides were labeled with ^33^P-dATP (PerkinElmer) using a three-terminal deoxynucleotidyl transferase enzyme kit (New England Biolabs). The reaction was conducted at 37°C for 2 h, and labeled oligonucleotides were purified with the QIAquick Nucleotide Removal Kit (QIAGEN). We used three different oligonucleotide mixes to hybridize to specific regions in the murine *Shank3* mRNA (NM_021423.4). The first mix corresponds to sequences located before the ankyrin domain (nucleotides 337-296, 413-372, and 483-440); the second mix corresponds to sequences within the ankyrin domain (nucleotides 653-609, 1106-1061, and 1254-1209, which are deleted in the *Shank3*^Δ*ex4-9*^ allele); the third mix corresponds to sequences after the ankyrin domain, within the proline-rich domain (nucleotides 3484-3440, 3693-3649, and 4127-4081). Prehybridation and hybridization conditions were performed as described by Julien et al. (2009). Hybridized, 12 µm brain sections were exposed to Kodak Biomax MR films for 20 d. Nonspecific hybridization was considered negligible, as determined by adding a 100-fold excess of unlabeled probes.

### Protein quantification using WB

Proteins from TBS-soluble, detergent-soluble, and PSD fraction of parietotemporal cortex and hippocampus were quantified by BCA protein assay, and equal amounts of total protein for each sample were added to Laemmli's loading buffer and heated 5 min at 95°C as described previously (Bourassa et al., 2019a). Ten to 20 µg of protein was resolved on a freshly prepared SDS-PAGE, and an electric field was applied. Membranes were probed overnight with the following synaptic and tau protein antibodies: anti-Shank3 (1:5000, Novus, NBP1-147610), anti-Shank3 (1:1000, Santa Cruz Biotechnology, SC-377088), anti-Actin (1:20,000, Abcam, abG043), anti-Cortactin (1:20,000, Abcam, 81208), anti-Drebrin (1:2500, Progen, MX823), anti-GFAP (1:5000, Sigma), anti-Homer 1 (1:2500, Santa Cruz Biotechnology, sc-136358), anti-Septin 3 (1:20,000, Novus, NBP1-56 101), anti-SNAP25 (1:20,000, Covance, SMI 81), anti-PSD-95 (1:60,000, NeuroMab, 75-028), anti-tau protein clone tau 13 (human) (1:5000, Covance), anti-total tau clone Tau C (1:10,000, Dako), anti-tau protein phosphorylated at Thr217 (1:1000, Invitrogen), anti-tau protein phosphorylated at Ser202/205 (clone CP13, 1:500, gift from Peter Davies), and anti-total tau clone Tau46 (1:1000, BioLegend). Chemiluminescence reagent was then added (Luminata Forte Western HRP substrate; Millipore) and the signal was imaged using the myECL Imager System (Fisher Scientific). Densitometric analysis was performed using the ImageLab 6 Software provided by Bio-Rad. Label-based LC/MS/MS quantification methods using isotopic labeling were used in collaboration with the Proteomics Core of the CHU de Quebec Research Center to compare the ratio of a given peptide across different samples of Shank3 WT and Shank3^Δex4-9^ mice. These data were used to determine the following proteins to investigate by WBs in our Shank3^Δex4-9^-3xTg-AD mice groups.

### Immunofluorescence

Immunofluorescence labeling was performed on vibratome 50-μm-thick sections from acrolein and PFA (Acrolein 3.5%, PFA 4%, both in PB, pH 7.4) perfused mice brain. Before immunostainning, floating sections were blocked with 5% NHS in 0.1 m PBS containing 0.4% Triton X-100 for 1 h. A polyclonal antibody targeting total Shank3 (PA5-77701, 1:500, Fisher Scientific) was use as primary antibody and detected with an anti-rabbit secondary antibody conjugated with AlexaFluor-647 (Invitrogen/Fisher Scientific). The nuclei were counterstained with DAPI (Pierce), and slides were incubated for 5 min in a 0.5% Sudan Black solution in 70% methanol to reduced autofluorescence cause by lipofuscin pigments. Slides were mounted using homemade Mowiol anti-fade mounting medium and dried overnight in the dark. Images of the Shank3 stained sections were recorded with EVOS FL Auto Imaging Systems (Fisher Scientific).

### Amyloid-β quantification by ELISA

Aβ40 and Aβ42 concentrations from soluble and detergent-insoluble fractions of parietotemporal cortex and hippocampal of 18-month-old Shank3^Δex4-9^-3xTg-AD mice were determined using highly sensitive ELISA kits according to the manufacturer's instructions (Wako), and plates were read at 450 nm using a Synergy HT multidetection microplate reader (BioTek) as previously described (Tremblay et al., 2007; Lebbadi et al., 2011; Vandal et al., 2016).

### Data and statistical analyses

When comparing two groups, the normality of data distribution within each group was assessed using the Shapiro–Wilk test. If the data distribution of either one or both groups failed to pass the normality test, groups were compared using a nonparametric Mann–Whitney test. Otherwise, an unpaired Student's *t* test was performed. When more than two groups were compared, nonparametric Kruskal–Wallis ANOVA followed by Dunn's multiple comparisons test or two-way ANOVA was used followed by a Sidak's multiple comparisons test. Significance of association between variables was determined by linear regression. The exact number of samples included are given in each figure legend. Each sample corresponds to an individual dot in the graphs. For behavioral tests in mice, each result has been obtained with at least a population of 21-41 mice in each group. For postmortem analysis, a minimum of 7 animals per group has been tested. Every experience has been realized with both sexes in approximately equal numbers. For all postmortem data, statistical significance was set at *p* < 0.05. Because of the relatively high statistical power for all behavioral tests, the threshold for significance was set at *p* < 0.01. Individual data were excluded for technical reasons if they did not meet preassigned conditions or if determined as an outlier. All statistical analyses were performed with Prism 9 (GraphPad) or JMP Statistical Analysis Software (version 15; SAS Institute).

## Results

### Loss of SHANK3a protein in AD brains and correlation with antemortem global cognitive score

**Figure 1.**
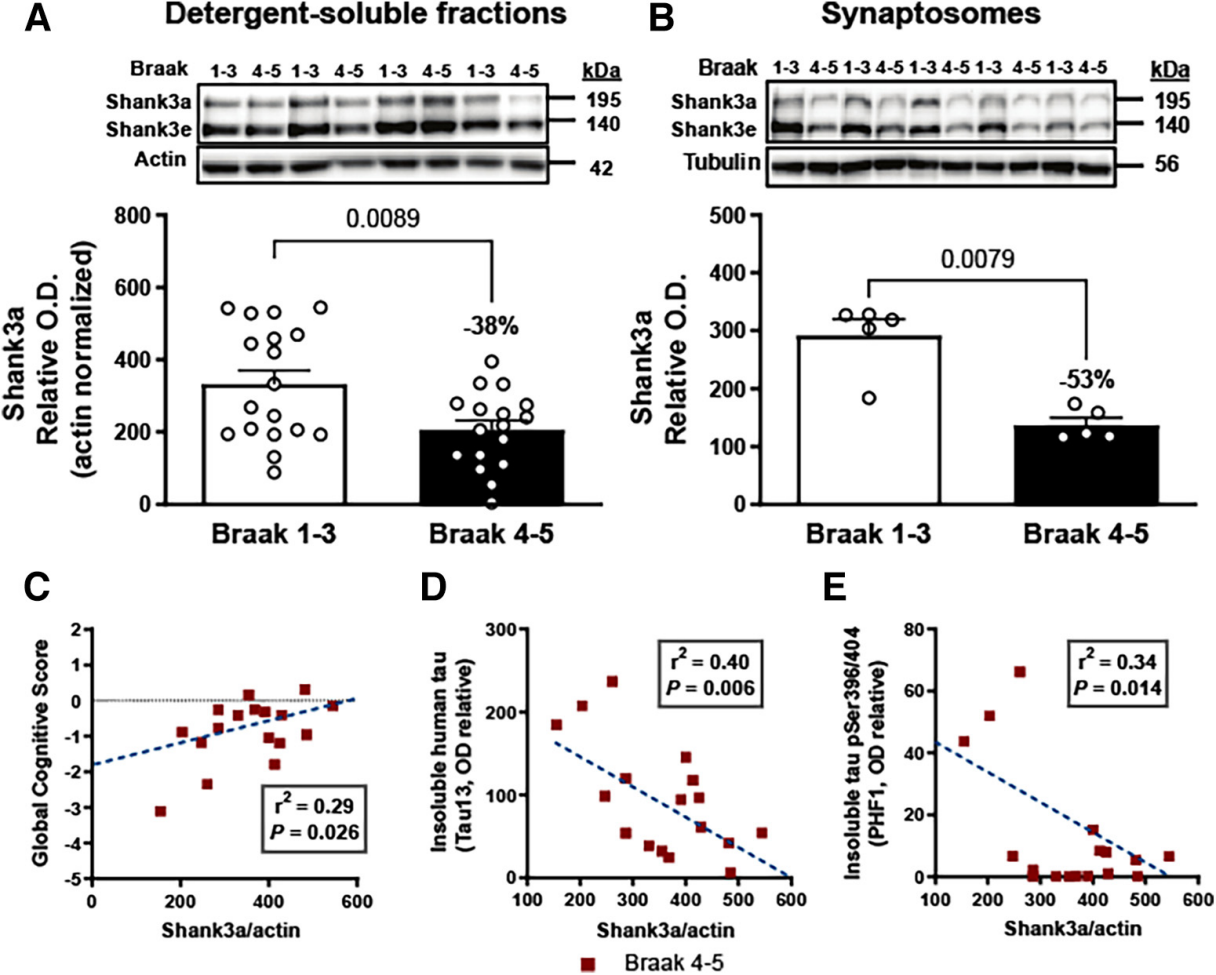
Shank3a loss in AD correlates with disease severity. ***A***, Shank3a (195 kDa) was quantified (antibody: ab136429) by WB in detergent-soluble extracts from parietal cortex of control (Braak Stages 1-3) and AD (Braak Stages 4 and 5) groups (*n* = 17 or 18). Shank3a levels were significantly reduced in individuals with AD. ***B***, The difference in Shank3a levels between controls and AD subjects was more marked in synaptosome extracts (*n* = 5). Linear regression analyses showed that Shank3a levels in the parietal cortex correlated (***C***) positively with global cognitive scores and (***D***) negatively with levels of insoluble total tau (Tau13) and (***E***) Insoluble phospho-tau labeled with PHF1 antibody in persons with AD. Data are mean ± SEM. *p* < 0.05. ***A***, ***B***, Data were compared using the Mann–Whitney test. ***C-E***, The coefficient of correlation *r*^2^ was calculated by linear regression. The *p* value was obtained using a generalized linear model.

First, we quantified SHANK3 in the detergent-soluble protein fractions from the parietal cortex of individuals with a neuropathological diagnosis of AD (Braak Stages 4 and 5), compared with controls (Braak Stages 1-3). We found significantly lower levels of SHANK3a protein (−38%, 195 kDa) in the parietal cortex from persons with a diagnosis of AD (Fig. 1*A*). Since SHANK3 is highly enriched in synapses, we also assessed the level of SHANK3 in synaptosome extracts from parietotemporal cortex and detected lower levels of SHANK3a (−53%) in individuals with a diagnosis of AD (Fig. 1*B*). More importantly, correlative analyses showed that SHANK3a was correlated with the global cognitive score (*r*^2^ = 0.29, *p* = 0.026) (Fig. 1*C*) in parietal cortex from people with AD.

Next, we explored the relationship between SHANK3 levels and other common neuropathological markers of AD in the same parietal cortex samples. Previous work with the same samples showed higher total-tau (tau 13 antibody) and pS396/404 tau (PHF1 antibody) levels in the parietotemporal cortex of AD patients (Tremblay et al., 2017). Here, inverse associations were found between SHANK3a and neurofibrillary tangles (*r*^2^ = 0.31, *p* = 0.02), insoluble total tau (*r*^2^ = −0.40, *p* = 0.006, Fig. 1*E*), and insoluble pS396/404 tau (*r*^2^ = −0.34, *p* = 0.014, Fig. 1*E*), suggesting that loss of SHANK3a is associated with an aggravation of tau pathology. No significant correlation was found with ELISA-determined concentrations of Aβ_42_ and Aβ_40_ in the soluble and detergent-insoluble protein fractions nor with neuritic plaques. Overall, these data suggest that the clinical and neuropathological progression of tau pathology is associated with a loss of SHANK3a.

### Generating a loss of Shank3a protein in 3xTg-AD mice

**Figure 2.**
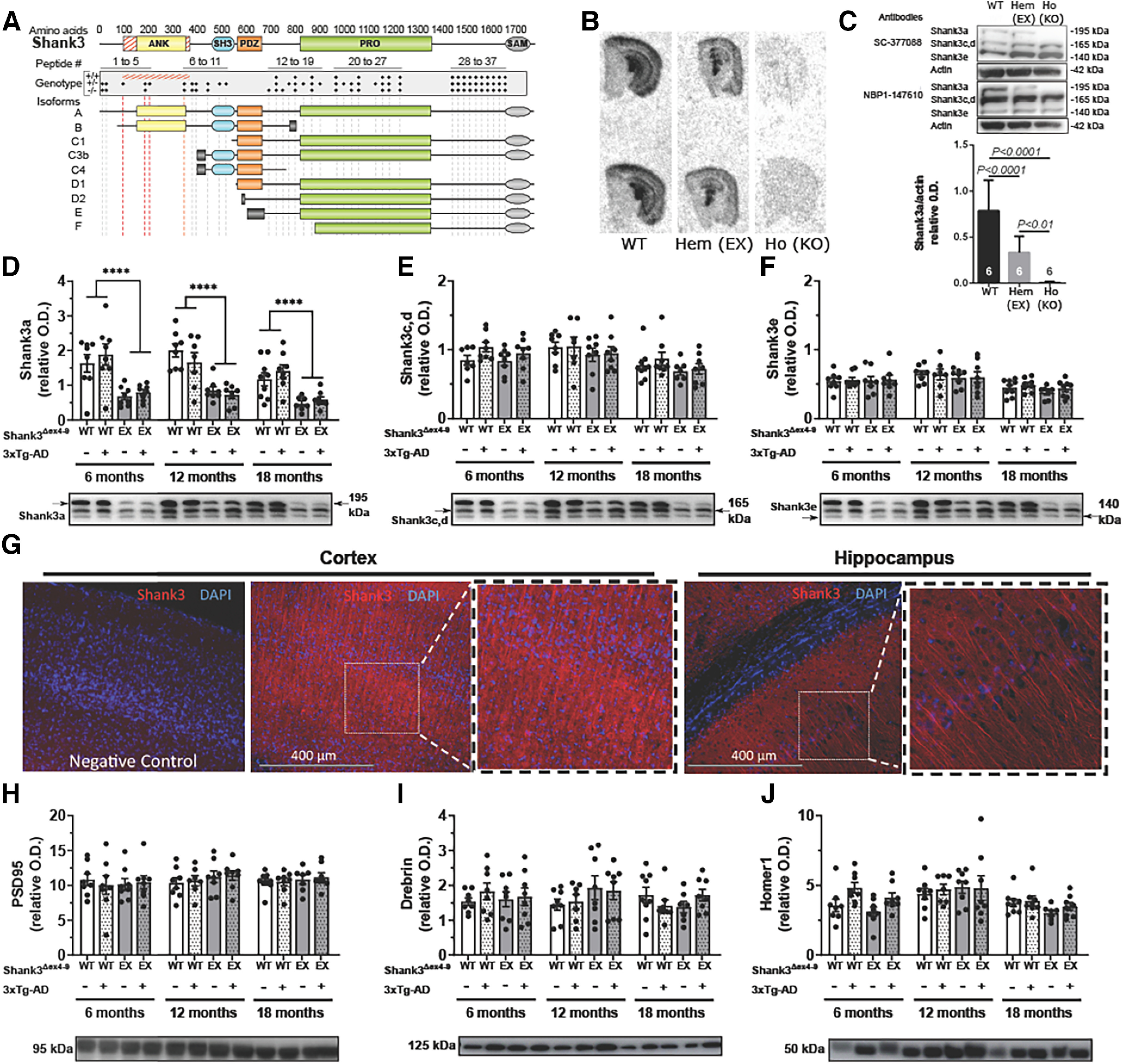
Characterization of Shank3a loss in the Shank3^Δex4-9^ and Shank3^Δex4-9^-3xTg-AD model. ***A***, Schematic representation of Shank3 and its five domains is shown relative to their amino acid position. Red hatched regions represent the deleted region of the *Shank3*^Δ*ex4-9*^ allele. Dotted red lines intersect with corresponding disrupted Shank3a and Shank3b isoforms, as confirmed by mass spectrometry. ***B***, In situ hybridization in coronal hemibrain sections using probes specific to exons 4-9 showed a decreased mRNA expression of Shank3a/Shank3b with the loss of one (Hem/EX) or two alleles (Ho/KO). ***C***, WB analysis of PSD fractions from the parietotemporal cortex of Shank3^Δex4-9^ mice using two C-terminal antibodies (SC-377088 and NPB1-147610) revealed an approximate decrease of Shank3a by 50% in hemizygous (Hem/EX) mice compared with WT mice, and a complete KO in homozygous (Ho/KO) mice, but no changes in isoforms Shank3c, Shank3d, and Shank3e. WB analyses targeting the 840-857 epitope (NBP1-147610) revealed a 50% reduction of (***D***) Shank3a, but normal levels of (***E***) Shank3c, Shank3d, and (***F***) Shank3e in PSD fractions of parietotemporal cortex of hemizygous Shank3^Δex4-9^ and Shank3^Δex4-9^-3xTg-AD mice at 6, 12, and 18 months of age. ***G***, Immunofluorescence analysis on brain coronal sections of WT mice using a pan-Shank3 antibody (PA5-77 701, epitope 841-855) showing signal in somatosensory cortex and CA1 region of the hippocampus. Insets, Higher magnification. Left, A negative control (10× magnification). No changes in postsynaptic proteins (***H***) PSD95, (***I***) drebrin, and (***J***) homer1 were detected in PSD fractions of parietotemporal cortex between each group of animals. Data are mean ± SEM (*n* = 5-9). *p* < 0.05. ****p < 0.0001, effect of Shank3 deletion (two-way ANOVA). ANK, Ankyrin; EX, ex4-9 excised from one allele; SH3, SRC homology 3; PRO, proline-rich; SAM, sterile α motif; Hem, hemizygous; Ho, homozygous; KO, knock-out.

Given the known impact of *SHANK3* haploinsufficiency, we hypothesized that Shank3 loss in the brain contributes to AD cognitive symptoms and neuropathology. We generated a novel model, the Shank3^Δex4–9^-3xTg-AD mouse, to study the effects of Shank3a depletion on AD neuropathology. In this model, a targeted disruption of *Shank3* was performed in which exons coding for the ankyrin repeat domain were deleted and expression of full-length *Shank3a* disrupted, as represented in Figure 2*A* (Bozdagi et al., 2010; Yang et al., 2012; Jiang and Ehlers, 2013). Since Shank3^Δex4-9^ consists of removing loxP-flanked exons 4 and 9, only *Shank3* isoforms containing these exons are affected by the mutation (Shank3a, Shank3b) (Yang et al., 2012). The result of this excision is a shift in the reading frame, leading to the insertion of a premature “Stop” codon and therefore the formation of an incomplete and nonfunctional Shank3a protein (Bozdagi et al., 2010; Yang et al., 2012). *Shank3* isoforms with translation initiation sites downstream of the deletion are unaffected.

ISH using probes that target sequences in the deleted exons 4-9 (nucleotides 653-609, 1106-1061, and 1254-1209) confirmed the loss of *Shank3a* mRNA in coronal brain sections of hemizygous and homozygous Shank3^Δex4-9^ mice, compared with controls (Fig. 2*B*). We next confirmed that the genetic ablation of Shank3a was translated at the protein level.

WB analysis showed a decrease of ∼50% of the Shank3a isoform in hemizygous Shank3^Δex4-9^ mice and a near-complete KO in homozygous mice (Fig. 2*C*). We then investigated Shank3a protein levels in the novel Shank3^Δex4-9^-3xTg-AD mouse model, compared with relevant groups. As expected, all mice with the Shank3^Δex4-9^ mutation showed a loss of Shank3a protein at each age tested (Fig. 2*D*). Levels of Shank3c, Shank3d, and Shank3e were also measured, but no difference was observed between groups for all ages, confirming the specificity of the Shank3^Δex4-9^ model (Fig. 2*E*,*F*). This result also confirmed that no intrinsic loss of Shank3 protein is found in 3xTg-AD mice, since no difference in Shank3 was observed between control and 3xTg-AD mice (Table 2). Still, a small reduction in Shank3 levels was detected with aging (Table 2). Immunohistofluorescence was used to visualize normal Shank3 distribution using pan-Shank3-specific antibodies (Fig. 2*G*). Shank3 immunosignal was localized in filaments and cell bodies in cortex and hippocampus (Fig. 2*G*). By contrast, concentrations of synaptic proteins known to interact with Shank3 (Drebrin, Homer, and PSD-95) remained unaffected by the partial Shank3a deletion in the same PSD fractions (Fig. 2*H–J*; Table 2). Other proteins probed and found to be unchanged by Shank3a depletion are shown in Table 2. Together, these results confirm the specific loss of the Shank3a isoform in Shank3^Δex4-9^ mice and that this decrease is maintained over time in this novel Shank3^Δex4-9^-3xTg-AD mouse model.

**Table 2.** Summary of synaptic protein quantification measured in postsynaptic densities by Western Blot*^a^*

Protein	Age (mo)	Shank3^WT^ NonTg [mean (SD); *n*]	Shank3^WT^ 3xTg-AD [mean (SD); *n*]	Shank3^Δex4-9^ NonTg [mean (SD); *n*]	Shank3^Δex4-9^ 3xTg-AD [mean (SD); *n*]	Kruskal–Wallis test (χ2)	Multivariate analyses
Shank3a	6	14.8 (6.4); 8	22.0 (14.6); 8	7.0 (3.1); 8	7.8 (2.2); 8	0.0032**	Lower in Shank3^Δex4-9^, *p* < 0.0001 Lower in older mice, *p* < 0.05
	12	19.7 (5.3); 8	16.7 (9.7); 7	7.7 (2.5); 8	6.2 (2.0); 8	0.0004***	
	18	11.3 (5.8); 9	13.5 (1.7); 8	4.3 (1.7); 7	5.2 (1.9); 8	0.0003***	
Shank3c, Shank3d	6	8.6 (2.2); 8	12.5 (7.3); 8	8.6 (2.9); 8	9.6 (3.6); 8	0.4127	Lower in older mice, *p* < 0.05
	12	10.3 (3.0); 8	10.6 (5.6); 7	8.5 (3.1); 8	8.5 (2.8); 8	0.5367	
	18	7.5 (2.2); 9	8.5 (1.3); 8	6.4 (2.3); 7	6.6 (2.3); 8	0.4326	
Shank3e	6	5.1 (1.3); 8	7.1 (5.0); 8	5.7 (2.6); 8	5.7 (2.4); 8	0.8728	Lower in older mice, *p* < 0.01
	12	6.5 (1.8); 8	6.4 (3.0); 7	5.5 (1.6); 8	5.3 (2.1); 8	0.5635	
	18	4.3 (1.0); 9	4.7 (0.6); 8	3.5 (1.3); 7	3.9 (1.3); 8	0.2001	
Shank1	6	3.6 (3.5); 8	4.8 (5.2); 8	4.2 (3.4); 7	2.7 (2.7); 8	0.8122	Lower in older mice, *p* < 0.05
	12	4.8 (6.6); 8	2.2 (2.9); 7	4.0 (3.6); 8	2.0 (1.7); 8	0.5598	
	18	2.0 (1.9); 9	1.6 (1.6); 8	1.2 (1.5); 7	1.9 (2.3); 8	0.8505	
Actin	6	2.3 (1.1); 8	2.7 (1.3); 8	2.1 (0.7); 8	2.0 (0.6); 8	0.6974	NS
	12	2.2 (0.5); 8	2.4 (0.6); 7	2.3 (0.7); 8	2.2 (0.7); 8	0.8678	
	18	2.5 (1.0); 9	2.6 (0.9); 8	2.6 (1.1); 7	2.8 (1.1); 8	0.9553	
Cortactin	6	2.8 (0.8); 8	3.6 (1.2); 8	2.9 (0.6); 8	3.1 (0.9); 8	0.5418	NS
	12	2.8 (0.7); 8	3.1 (0.6); 7	2.8 (0.5); 8	2.9 (0.5); 8	0.8895	
	18	2.8 (0.7); 9	3.0 (0.4); 8	2.4 (1.0); 7	3.0 (1.0); 8	0.3829	
Drebrin	6	1.5 (0.5); 8	2.1 (1.1); 8	1.7 (0.8); 8	1.7 (0.9); 8	0.6974	NS
	12	1.4 (0.5); 8	1.5 (0.7); 7	1.8 (1.1); 8	1.6 (0.6); 8	0.9219	
	18	1.6 (0.6); 9	1.4 (0.4); 8	1.3 (0.4); 7	1.6 (0.6); 8	0.3297	
GAP-43	6	4.5 (1.8); 8	6.0 (3.3); 8	5.2 (2.4); 8	5.1 (2.0); 8	0.8795	NS
	12	5.0 (3.0); 8	4.4 (2.7); 7	5.6 (2.6); 8	4.8 (2.6); 8	0.7553	
	18	3.8 (1.7); 9	3.9 (1.9); 8	3.8 (1.2); 7	3.9 (2.0); 8	0.9956	
Homer 1	6	3.3 (0.7); 8	4.6 (0.8); 7	3.2 (1.1); 8	4.0 (0.8); 8	0.0200*	Higher in 3xTg-AD, *p* < 0.01
	12	4.4 (1.2); 8	4.6 (1.7); 7	4.4 (1.3); 8	4.2 (1.9); 8	0.8028	
	18	3.6 (0.6); 9	3.8 (1.1); 8	2.8 (0.6); 7	3.1 (0.7); 8	0.0872	
Septin 3	6	4.8 (1.5); 8	6.5 (3.2); 8	5.1 (1.4); 8	5.4 (1.7); 8	0.6895	NS
	12	6.0 (1.7); 8	6.1 (2.1); 7	5.7 (1.7); 8	5.8 (1.8); 8	0.9779	
	18	5.4 (1.4); 9	5.7 (1.2); 8	5.2 (1.5); 7	5.3 (1.5); 8	0.8709	
SNAP25	6	3.7 (1.6); 8	5.0 (3.8); 8	3.8 (1.6); 8	3.4 (1.2); 8	0.7041	NS
	12	3.9 (1.5); 8	4.2 (1.6); 7	3.8 (1.5); 8	3.5 (1.2); 8	0.8749	
	18	3.7 (1.2); 9	4.0 (1.5); 8	3.7 (1.7); 7	3.6 (1.7); 8	0.9131	
Spectrin	6	2.4 (1.2); 8	3.0 (2.4); 8	2.5 (1.1); 8	2.3 (0.9); 8	0.9538	Lower in older mice, *p* < 0.05
	12	1.8 (0.6); 8	1.5 (0.5); 7	1.8 (0.6); 8	1.9 (0.8); 8	0.6661	
	18	2.1 (0.6); 9	2.1 (0.6); 8	1.9 (0.7); 7	2.1 (0.8); 8	0.8514	
Synaptophysin	6	8.3 (5.2); 8	14.6 (10.0); 8	7.7 (5.7); 8	12.2 (9.3); 8	0.3200	Higher in 3xTg-AD, *p* < 0.05
	12	6.3 (3.2); 8	12.1 (11.6); 7	8.5 (3.6); 8	7.8 (4.1); 7	0.7291	
	18	11.7 (5.9); 9	12.9 (3.7); 8	9.3 (5.6); 7	14.0 (10.9); 8	0.7402	
PSD95	6	10.8 (2.3); 8	10.0 (3.8); 8	10.2 (2.4); 8	10.5 (2.7); 8	—	—
	12	10.3 (2.2); 8	10.7 (2.0); 7	11.2 (2.4); 8	11.4 (1.9); 8	—	
	18	10.5 (1.3); 9	10.5 (1.7); 8	10.8 (1.7); 7	11.2 (1.7); 8	—	

*^a^*Synaptic protein levels from the PSD fraction of parietotemporal cortex of Shank3^Δex4-9^-3xTg-AD mice were measured by WB. Data were normalized with PSD95 and are mean ± SD followed by the sample size of each group. GAP-43, Growth-associated protein 43; SNAP-25, synaptosomal-associated protein 25 kDa; PSD-95, PSD protein 95.

Statistical analysis: nonparametric Kruskal–Wallis test,

*p < 0.01,

**p < 0.001,

***p < 0.0001; multivariate analysis (for age, Shank3^Δex4-9^, and 3xTg-AD genotypes) is also shown.

### Shank3 protein deficiency aggravates cognitive impairments of 3xTg-AD mice

**Figure 3.**
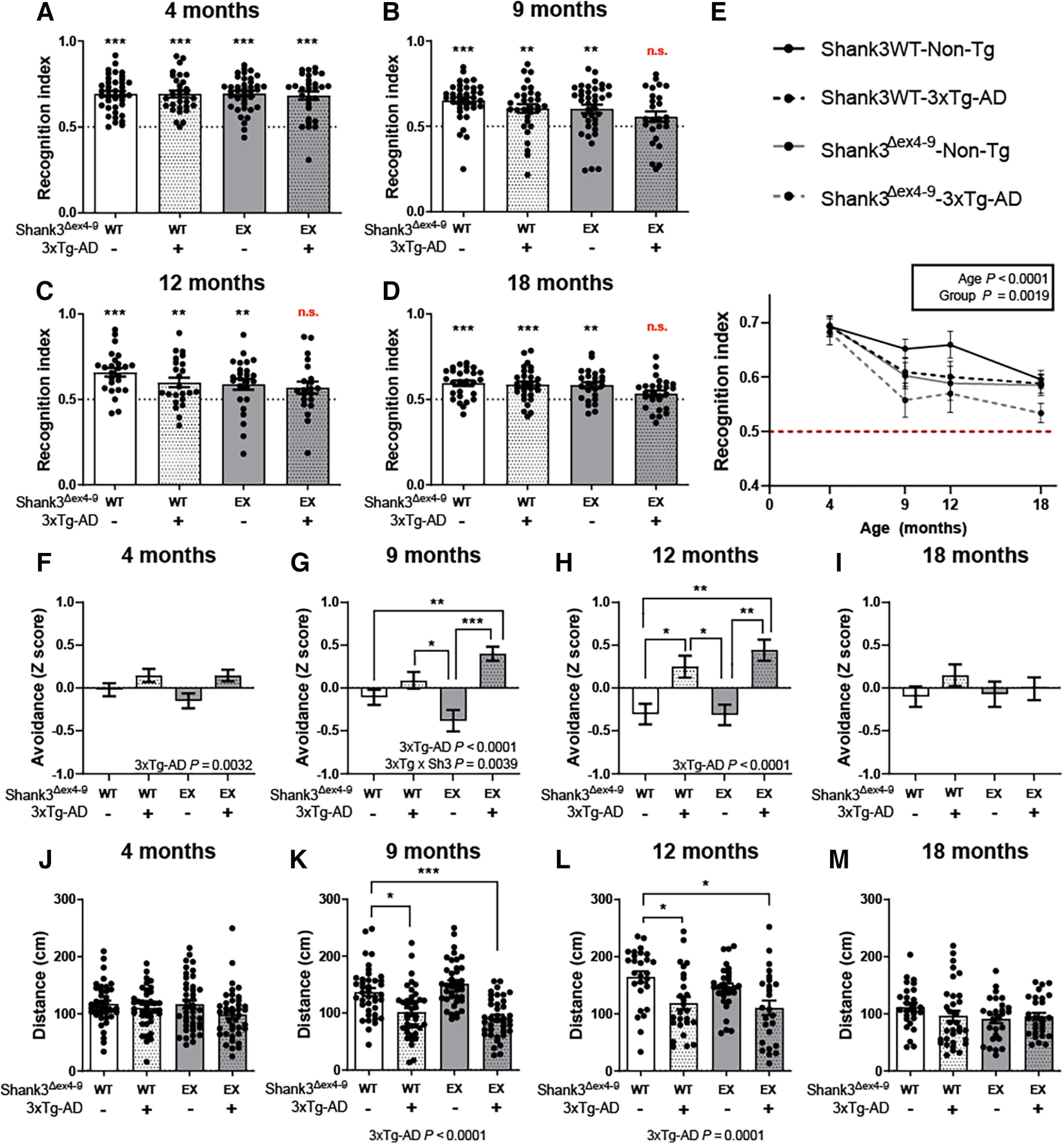
Shank3a deficiency showed a synergistic effect with AD-like pathology on recognition memory and anxiety-like behavior in hemizygous 3xTg-AD mice. ***A-E***, NOR test was performed on hemizygous Shank3^Δex4-9^-3xTg-AD mice of 4, 9, 12, and 18 months of age (*n* = 25-39 per group). Only 9-, 12-, and 18-month-old Shank3^Δex4-9^-3xTg-AD mice failed to interact with the new object significantly more than with the familiar one, consistent with an apparent synergy between Shank3a disruption and APP/tau transgenes detectable after 9 months. ***F-I***, Shank3a deficiency aggravated anxiety-related impairments in hemizigous 3xTg-AD mice as early as 9 months of age, whereas those with normal levels of Shank3a did not show impairment until 12 months of age (*n* = 25-41 per group). No difference was observed in 18-month-old mice. ***J-M***, No difference in exploratory behavior in 4- and 18-month-old mice was observed during the open field test, but the 3xTg-AD genotype was associated with shorter distance traveled at 9 and 12 months of age (*n* = 25-41 per group). Data are mean ± SEM. *p* < 0.01. ***A-D***, Data were compared using one-sample *t* test versus theoretical mean of 0.5, where significance indicates successful object recognition. ***E***, Data were compared using two-way ANOVA for age and group variables. ***F-M***, Data were compared using a two-way ANOVA for 3xTg-AD and Shank3^Δex4-9^ genotypes and a Kruskal–Wallis test followed by Dunn's test. *p < 0.01. ** *p* < 0.001. *** *p* < 0.0001. EX, ex4-9 excised from one allele.

Behavioral tests were performed to determine the impact of Shank3 deficit on recognition memory, anxiety, and locomotion in mice over time. The 3xTg-AD mouse displays impaired learning and memory and anxiety-like behavior (Sterniczuk et al., 2010; St-Amour et al., 2014; Vandal et al., 2015; Dal-Pan et al., 2017). To determine whether Shank3 deficit translated into memory defects, all groups of mice underwent the NOR test at 4, 9, 12, and 18 months of age (St-Amour et al., 2014; Vandal et al., 2015). The RI was calculated as the time spent interacting with the novel object divided by the total time of exploration during the testing phase. As random exploration of the two objects would result in an RI of 0.5, we analyzed the statistical difference between this theoretical value and the mean value of each group. Four-month-old mice were successful in the identification of the novel object, consistent with an RI significantly higher than 0.5 (Fig. 3*A*). At 9 months of age, control, Shank3-deficient, and 3xTg-AD mice were still able to recognize the new object. However, Shank3^Δex4-9^-3xTg-AD mice failed to distinguish the novel object from the old one. While a decline in the RI was detected with age (Fig. 3*E*), this pattern was preserved in 12- and 18-month-old mice as only Shank3^Δex4-9^-3xTg-AD mice still failed to recognize the novel object (Fig. 3*C–E*). Since both independent variables taken separately did not impair memory at 9, 12, or 18 months of age, these data suggest a synergistic deleterious effect of the Shank3 deficiency and the 3xTg-AD genotype on recognition memory (Fig. 3*A–E*).

The light & dark box and open field behavioral tests were performed to assess anxiety and exploratory behavior. Avoidance scores were used to estimate the aversion of the mice for stressful areas (illuminated chamber and center of the chamber in light & dark box and open field test, respectively). A variation of a composite avoidance score was calculated as described previously (Sterniczuk et al., 2010; Toth et al., 2016; Deslauriers et al., 2017). Two-way ANOVA showed that the aggravating effect of the 3xTg-AD transgenes on this anxiety-related behavior was significant at 4, 9, and 12 months of age (Fig. 3*F–H*) but lost at 18 months (Fig. 3*I*). This difference was more robust at 9 and 12 months where the avoidance composite score was significantly higher in 3xTg-AD mice compared with control or Shank3^Δex4-9^ hemizygous (Fig. 3*G*,*H*). At 9 months of age, Shank3 deficiency combined with 3xTg-AD transgenes displayed additive effects on the anxiety-like behavior, while at 12 months the expression of 3xTg-AD transgenes on one allele was sufficient to induce the change (Fig. 3*G*,*H*). Locomotion was also assessed in open field test. No difference was found between groups of 4-month-old mice (Fig. 3*J*). However, 9- and 12-month-old mice expressing the 3xTg-AD transgenes traveled less than other groups of the same age. This reduced exploratory activity in 3xTg-AD mice is consistent with previous work (Halagappa et al., 2007; Nelson et al., 2007; Arsenault et al., 2011; Bories et al., 2012). At 18 months of age, locomotor activity was low and similar between groups. No significant association between PSD levels of Shank3a and behavioral endpoints was detected. These results indicate that changes in voluntary locomotor activity or exploratory behavior cannot explain the changes in performance observed in the NOR test.

### Shank3a protein deficiency increases levels of Aβ42 peptide and soluble tau

**Figure 4.**
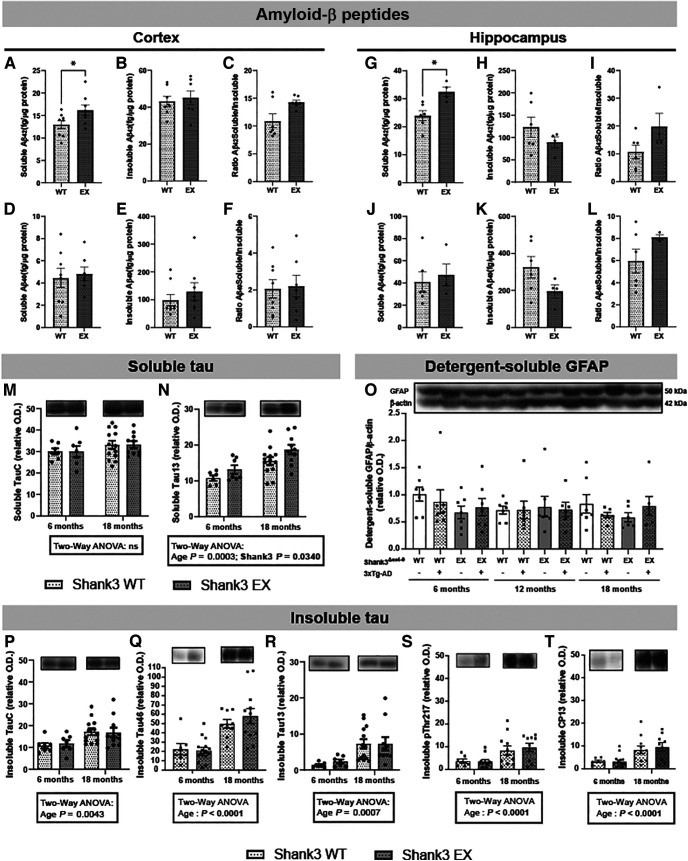
Shank3a deficiency increased soluble Aβ42 and tau accumulation in 18-month-old hemizygous 3xTg-AD mice. Soluble and insoluble Aβ42 (***A-C***,***G-I***) and Aβ40 (***D-F***,***J-L***) peptides were measured by ELISA in the parietotemporal cortex (***A-F***) and hippocampus (***G-I***) of hemizigous 3xTg-AD females. Shank3a deficiency increased levels of soluble Aβ42 in female 3xTg-AD mice in the cortex (***A***) and hippocampus (***G***). By contrast, concentrations of insoluble Aβ42, soluble Aβ40, and insoluble Aβ40 were not significantly different between groups in either brain region. Levels of total (human + murine) tau (Tau C, ***M-P***; Tau46, ***Q***), human tau (Tau 13, ***N-R***), and phosphorylated tau (pThr217, ***S***; CP13, ***T***) were measured by WB in the parietotemporal cortex of male and female hemizigous 3xTg-AD mice. Soluble human tau levels were increased by the loss of Shank3a and by aging (***N***). Insoluble total, human and phosphorylated tau levels were higher in 18-month-old animals but were not altered by Shank3a deficiency (***P-T***). Detergent-soluble GFAP levels were not altered by Shank3a deficiency neither by age (***O***). Representative bands were selected from non-consecutive samples run on the same gel. Data are mean ± SEM. Representative bands were selected from nonconsecutive samples run on the same gel. Data are mean ± SEM. ***A-L***, *p < 0.05 (unpaired Student's *t* tests). ***M-T***, Data are compared using two-way ANOVA. EX, ex4-9 excised from one allele.

The use of the 3xTg-AD mouse brings the possibility of determining whether Shank3a loss affects the main neuropathological markers of AD, namely, Aβ and tau pathologies. We thus quantified Aβ and tau in the soluble and insoluble fractions of parietotemporal cortex and hippocampus of 18-month-old 3xTg-AD mice with or without concomitant Shank3^Δex4-9^ mutation. Figure 4 and Table 3 present ELISA results of both Aβ_42_ and Aβ_40_ in the soluble and detergent-insoluble protein fraction (aggregated Aβ peptides) of parietotemporal cortex and hippocampus in 18-month-old mice, an age at which Aβ is present in high enough concentrations for valid quantification in the cortex and hippocampus of hemizygous 3xTg-AD mice (Lebbadi et al., 2011; Arsenault et al., 2013). Data were analyzed separately for males and females since Aβ pathology differentially affects females compared with males in 3xTg-AD model (Bories et al., 2012; Vandal et al., 2015). In females, Shank3a deficiency led to higher amounts of soluble Aβ_42_ peptides in both the parietotemporal cortex (Fig. 4*A*, *p* = 0.0494) and the hippocampus (Fig. 4*G*, *p* = 0.0119) of 3xTg-AD mice, whereas insoluble Aβ_42_ and Aβ_40_ remained unchanged (Fig. 4*D–F*,*J*,*L*). No change in Aβ pathology was observed in males (Table 3). Changes in Aβ_42_/_40_ ratio remained nonsignificant (Fig. 4; Table 3).

**Table 3.** Quantification of soluble and insoluble amyloid-β in parietotemporal cortex and hippocampus of 3xTg-AD mice*^a^*

	Cortex [mean (SEM)]	3xTg-AD	Shank3^Δex4-9^-3xTg-AD	*p*
Soluble fraction (fg/μg of total protein)	Males			
	Amyloid-β42	3.91 (0.83)	2.65 (0.19)	0.1893
	Amyloid-β40	1.79 (0.33)	2.24 (0.46)	0.4384
	Ratio amyloid-β42/40	2.48 (0.48)	1.46 (0.34)	0.1417
	Females			
	Amyloid-β42	12.96 (0.93)	16.19 (1.18)	0.0494*
	Amyloid-β40	4.45 (0.88)	4.82 (0.61)	0.7469
	Ratio amyloid-β42/40	3.29 (0.80)	3.57 (0.51)	0.7776
Insoluble fraction (fg/mg of total tissue)	Males			
	Amyloid-β42	30.83 (2.08)	28.59 (0.62)	0.3341
	Amyloid-β40	72.79 (15.37)	71.52 (11.93)	0.9497
	Ratio amyloid-β42/40	0.41 (0.11)	0.54 (0.13)	0.4958
	Females			
	Amyloid-β42	43.22 (2.93)	45.17 (3.75)	0.6884
	Amyloid-β40	98.15 (20.96)	128.69 (31.94)	0.4418
	Ratio amyloid-β42/40	0.61 (0.10)	0.52 (0.07)	0.4862

	Hippocampus [mean (SEM)]	3xTg-AD	Shank3^Δex4-9^-3xTg-AD	*p*
Soluble fraction (fg/μg of total protein)	Males			
	Amyloid-β42	12.31 (1.32)	11.04 (0.67)	0.4114
	Amyloid-β40	34.06 (8.39)	39.66 (6.21)	0.6036
	Ratio amyloid-β42/40	0.48 (0.11)	0.33 (0.09)	0.4848
	Females			
	Amyloid-β42	23.95 (1.81)	32.46 (1.68)	0.0119*
	Amyloid-β40	40.87 (8.92)	47.37 (9.63)	0.6431
	Ratio amyloid-β42/40	0.71 (0.14)	0.72 (0.09)	0.9480
Insoluble fraction (fg/mg of total tissue)	Males			
	Amyloid-β42	76.32 (19.53)	66.63 (15.94)	0.7088
	Amyloid-β40	240.52 (74.33)	156.34 (64.97)	0.2343
	Ratio amyloid-β42/40	0.25 (0.04)	0.53 (0.12)	0.0630
	Females			
	Amyloid-β42	123.39 (22.87)	89.29 (12.07)	0.2907
	Amyloid-β40	325.29 (57.75)	195.39 (35.28)	0.1318
	Ratio amyloid-β42/40	0.37 (0.02)	0.46 (0.03)	0.0433*

*^a^n* = 4-8 per group. Measurements of Aβ_40_ and Aβ_42_ were performed by ELISA. Data are mean ± SEM.

Statistical analysis: compared with 3xTg-AD mice without *Shank3* deletion within each corresponding tissue, fraction, and sex groups, unpaired *t* test, two-tailed,

*p < 0.05.

We further investigated the effect of a loss of Shank3a on the accumulation of total tau (Tau C and Tau46) and human tau (Tau 13) on both male and female 3xTg-AD mice. The Tau 13 antibody specifically detects human tau protein from the transgene, while Tau C and Tau46 antibodies reveal both human and mouse tau in brain samples. We also probed for phosphorylated tau at epitopes 202/205 and 217. As expected, older age was associated with high levels of soluble human tau and with more prominent accumulation of insoluble tau (Fig. 4*N*,*P–T*). The loss of Shank3a induced a small but significant increase in soluble human tau (*p* = 0.0340, Fig. 4*N*) but had no significant effect on other tau species investigated (Fig. 4*M*,*P–T*). Finally, no change in glial activation was detected between groups, as assessed with the levels of GFAP in parietotemporal cortex of mice (Fig. 4*O*).

## Discussion

The present study investigated whether Shank3 in the brain contributes to AD cognitive symptoms and neuropathology. The implication of the *SHANK3* gene in cognitive dysfunction has been confirmed in neurodevelopmental pathologies. Yet, few have investigated its role in neurodegenerative diseases. The present investigation is consistent with the following conclusions: (1) lower levels of Shank3 are found in the parietal cortex of individuals with AD, correlating with cognitive decline and tau accumulation; (2) a specific Shank3a deficit on one allele can be replicated in an animal model of AD; (3) the partial Shank3a loss synergizes with Aβ and tau neuropathologies to induce impairment in object recognition and anxiety-like behavior; and (4) defects in Shank3a increase the concentrations of transgene-induced Aβ42 and tau in the cortex of 3xTg-AD mice.

The diagnosis of AD based on Braak criteria was associated with ∼40% lower SHANK3a concentrations in the parietal cortex, in agreement with previous immunoblotting and immunostaining analyses performed in the cortex and hippocampus of AD patients (Gong et al., 2009; Pham et al., 2010). The difference with controls was also found in synaptosome extracts from a different cohort, consistent with a synaptic localization of this defect. Importantly, such a magnitude of decrease in SHANK3 in the brain has been shown to cause severe mental retardation in children with Phelan–McDermid syndrome (Grabrucker et al., 2011a). Although no association was found between age of death and SHANK3 levels, an earlier AD diagnosis was significantly associated with lower brain SHANK3a concentrations, suggesting that the loss of SHANK3 may characterize a more aggressive form of the disease. The Religious Orders Study allowed us access to antemortem cognitive performance data and detailed neuropathology (Bennett et al., 2012; Tremblay et al., 2017). We found a significant positive association between cortical SHANK3a levels and global cognitive scores. We also detected significant associations between SHANK3a loss and the accumulation of total tau and phosphorylated tau in the detergent-insoluble extracts from the parietal cortex. These associations were found within persons with AD, consistent with a loss of SHANK3a along with the progression of the disease. Our results indicate that a decrease in SHANK3a concentrations in the parietal cortex is associated with both clinical symptoms and tau neuropathology in AD. Based on the evidence gathered from the known impact of SHANK3 haploinsufficiency, these results suggest that such a SHANK3 protein deficiency could contribute to AD cognitive symptoms.

We next generated an animal model lacking Shank3a on one allele and bearing Aβ and tau pathology-inducing transgenes on the other allele. The specific decrease of ∼50% of Shank3a was established using WB and ISH. The wide range of experiments conducted here confirm the validity of such a Shank3^Δex4-9^-3xTg-AD model for the investigation of the Shank3a deficiency in a murine model of AD. The extent of the loss of Shank3a observed in this mouse model was similar to what we found in AD and to what is observed in children with Phelan–McDermid syndrome, but was not accompanied by changes in other synaptic proteins levels. Given the central role of Shank3 in PSD scaffold (Naisbitt et al., 1999; Roussignol et al., 2005; Boeckers, 2006; Grabrucker et al., 2011a; Raynaud et al., 2013; Dosemeci et al., 2016), it could have been expected that a loss of Shank3a would disrupt the PSD complex, and lead to the release of other PSD-related proteins. We probed for several scaffolding proteins, such as PSD-95, drebrin, septin, and homer, and found no change. As many of these postsynaptic proteins are reported to be lower in AD brain (Calon et al., 2004; Scheff et al., 2014; Forner et al., 2017; Tremblay et al., 2017), this would have provided a cogent explanation for the synergy observed in cognitive impairment. However, it is possible that the loss of *SHANK3a* from one allele induced dysfunction of PSD without global loss of scaffolding proteins. For example, a redistribution of these proteins leading to deleterious changes in function may occur without being detected in PSD homogenates. It should also be noted that the present mouse model specifically lacks half of *Shank3a*, but other isoforms were unaffected by the partial deletion. Different results may have been obtained using a model lacking all *Shank3* isoforms (Drapeau et al., 2018), which would have prevented possible compensation mechanisms by these other isoforms. In any case, the present work indicates that a specific Shank3a deficit can be replicated in an animal model of AD.

A notable observation is that Shank3 defects synergized with APP/tau transgenes to impair object recognition and precipitate anxiety-like behavior. Both Shank3^Δex4-9^ and 3xTg-AD mice have been shown to display several behavior anomalies in previous publications. Because of its key role in autism spectrum disorder, Shank3 mice have chiefly been investigated at a young age. The most common defects reported in hemizygous Shank3-deficient mice are defects in social interaction domains (Bozdagi et al., 2010, 2013; Drapeau et al., 2014; Jaramillo et al., 2016; Monteiro and Feng, 2017). Given the *SHANK3* haploinsufficiency in humans, it was imperative here to use hemizygous animals to investigate a 50% loss, closer to what we observed in AD brain. Here, the loss of *Shank3a* on a single allele did not induce significant effect on object recognition, emergence from a dark compartment, or locomotion. The 3xTg-AD mouse, by contrast, is typically used at an older age with homozygous breeds (Oddo et al., 2003; St-Amour et al., 2014; Vandal et al., 2015). Using hemizygous 3xTg-AD mice as reported here leads to the limited AD-like neuropathology and behavior, facilitating the study of synergies with aggravating factors (Lebbadi et al., 2011; Arsenault et al., 2013; Bories et al., 2017; Virgili et al., 2018). We observed that mice expressing the 3xTg-AD transgenes on a single allele traveled less distance at the age of 9 and 12 months, in agreement with a previous study with the hemizygous 3xTg-AD mouse (Bories et al., 2017), but such a difference was not observed in another study (Virgili et al., 2018). The AD transgenes alone also slightly aggravated anxiety readout at 12 months, but not at the other ages. Interestingly, neither *Shank3a* defects nor AD transgenes alone had any significant impact on object memory. The concomitant loss of *Shank3a* and AD transgene expression was necessary for the apparition of cognitive impairments at 9, 12, and 18 months of age. While the data show a significant decrease with age of the RI in all animals, Shank3^Δex4-9^-3xTg-AD mice were the only group which failed the test at 9, 12, and 18 months, despite a relatively high statistical power to detect a difference from a random choice. Since Shank3a deficiency and APP/tau transgenes alone could not generate such a defect, our results are consistent with a synergy between both factors. Shank3^Δex4-9^-3xTg-AD mice also displayed earlier anxiety-related behavior on two anxiety-related tasks, suggesting a synergistic effect on this AD-relevant behavior as well. This set of behavioral results suggests that synaptic defects occurring in AD, and here exemplified by a reduction in Shank3a, may synergize with classical Aβ/tau neuropathologies to aggravate the clinical mnemonic and anxiety symptoms of AD.

The present study delivers some clue about whether the defect in SHANK3a may be upstream or downstream of AD neuropathology. While the analyses in human brain are only correlative, the animal models can help determine the impact of each variable separately. First, we did not observe any significant change in Shank3a levels in 3xTg-AD mice at 6, 12, and 18 months of age. This suggests that the reduction in SHANK3a in AD is not just a consequence of Aβ and tau pathologies. However, there was a small age-related decrease in Shank3a levels in the mouse. These results allowed us to determine the independent effect of Shank3a on Aβ and tau pathology. By contrast, the genetically induced loss of *SHANK3a* on a single allele appeared to exert an effect on Aβ and tau concentrations in the 3xTg-AD model. The more consistent change observed was higher levels of soluble Aβ in both the hippocampus and the parietotemporal cortex of Shank3^Δex4-9^-3xTg-AD mice. The defects in Shank3a also led to higher soluble tau in the parietotemporal cortex, when assessed with an antibody specific for human tau. The specificity of this effect to the human tau and not to the murine tau is of interest because murine tau does not aggregate as human tau does, and only human tau forms PHF and neurofibrillary tangles (Kampers et al., 1999; Binder et al., 2005; Maeda et al., 2007; Xu et al., 2014; Ontiveros-Torres et al., 2016). The present data therefore indicate that Shank3a may play a role in the accumulation of Aβ and tau at least in their soluble form. Overall, these results suggest that the loss of Shank3a does not appear to be a secondary consequence of Aβ/tau neuropathology, and may rather occur before or in parallel with the accumulation of classical AD neuropathology.

Previous studies have shown that Aβ oligomers bind to Shank3 (Ding et al., 2019) and that the addition of Aβ40 or Aβ42 in the micromolar range in the media lowers Shank1 and Shank3 in synapses formed by neurons in culture (Roselli et al., 2009; Grabrucker et al., 2011b), an effect that is reversed by the addition of zinc (Grabrucker et al., 2011b; Grabrucker, 2014). Given the suspected link between zinc deficiency and AD, lower brain levels of zinc could underlie the specific reduction in Shank3a observed here (Baum et al., 2010; Grabrucker, 2014; Kim et al., 2021; Rivers-Auty et al., 2021; Chen et al., 2023) and the effect of Shank3a deficiency on tau and Aβ could form an amplification loop in AD. However, adding one copy of 3xTg-AD transgenes to WT or hemizigous Shank3^Δex4-9^ mice to induce Aβ pathology did not lead to a clear decrease of Shank3 in the present study, even in females with higher Aβ levels. It is possible that, in the case of hemizygous 3xTg-AD mice, cerebral levels of Aβ and Zn^2+^ do not reach the threshold necessary to dislocate Shank3 scaffold.

In conclusion, the human and animal data included in this report show that a loss of Shank3a is associated with AD, in which it may synergize with Aβ and tau neuropathology to induce memory defects. Most notably, transgenic deactivation of Shank3a on a single allele in the brain slightly enhanced brain Aβ/tau pathologies and produced behavior deficits only in animals already expressing AD transgenes. To our knowledge, Shank3 is the only synaptic protein that shows haploinsufficiency. These results thus suggest that specifically preventing the deficit in SHANK3 in the synapses of AD patients may prove beneficial against cognitive symptoms of AD.
